# *Weizmannia coagulans* SA9: A Novel Strategy to Alleviate Type 2 Diabetes

**DOI:** 10.3390/nu17132081

**Published:** 2025-06-23

**Authors:** Linhao Wang, Jie Wang, Yewei Tan, Changyu Cai, Xiaohua Yang, Sashuang Dong, Jiaqi Hong, Xiang Fang, Hong Wei, Zhenlin Liao

**Affiliations:** 1 College of Food Science, South China Agricultural University, Guangzhou 510642, China; wang_lin_hao@163.com (L.W.); jiewang@scau.edu.cn (J.W.); caichangyu122@163.com (C.C.); y-xiaohua@stu.scau.edu.cn (X.Y.); dongsashuang@126.com (S.D.); jiaqi@stu.scau.edu.cn (J.H.); fxiang@scau.edu.cn (X.F.); 2 College of Animal Sciences and Technology, Huazhong Agricultural University, Wuhan 430070, China; tyw13135426947@163.com

**Keywords:** *Weizmannia coagulans*, type 2 diabetes, 1-deoxynojirimycin, gut microbiota

## Abstract

**Background**: Probiotics have recently emerged as promising agents in the prevention and treatment of various human diseases. This study investigates the therapeutic potential of *Weizmannia coagulans* SA9 in the management of type 2 diabetes mellitus (T2DM). **Methods**: The in vitro antidiabetic activity of *W. coagulans* SA9 was primarily assessed via its α-glucosidase inhibitory capacity, complemented by metabolomic profiling to identify putative bioactive metabolites. The antidiabetic efficacy was further evaluated in a db/db mouse model, focusing on glucose tolerance, inflammatory biomarkers, and gut microbiota composition. **Results**: *W. coagulans* SA9 showed significant inhibitory effects on α-glucosidase and α-amylase, and DNJ and other active substances were detected in its culture supernatant. After 6 weeks of continuous administration, the fasting blood glucose level, glucose tolerance, and inflammation indexes of mice were significantly improved. Beneficial changes in the structure of the intestinal flora occurred after the probiotic intervention, as evidenced by a significant decrease in harmful bacteria (e.g., *Aerococcus urinaeequi*) and a significant enrichment of beneficial bacteria (e.g., *Limosilactobacillus reuteri*). **Conclusions**: *W. coagulans* SA9 exerts robust antidiabetic effects and holds promise as a novel strategy for the prevention and management of T2DM.

## 1. Introduction

In recent years, the prevalence of chronic metabolic diseases—particularly diabetes—has risen dramatically, largely driven by shifts in lifestyle patterns such as the adoption of high-sugar, high-fat diets, increased consumption of processed foods, and insufficient physical activity. According to the International Diabetes Federation (IDF), the global diabetic population surpassed 537 million in 2021 and continues to increase at an alarming pace, representing a major public health concern [1]. Diabetes is a metabolic disorder characterized primarily by hyperglycemia, resulting from insulin resistance or inadequate insulin secretion [2]. Type 2 diabetes mellitus (T2DM), also referred to as non-insulin-dependent diabetes, constitutes more than 90% of all diabetes cases [3]. Key risk factors for T2DM include obesity, unhealthy dietary patterns, physical inactivity, genetic predisposition, and advancing age. Middle-aged and older adults, as well as individuals with a family history of diabetes, are at particularly high risk. The most significant health risks associated with diabetes stem from its long-term complications, including diabetic nephropathy, diabetic foot, and retinopathy. These complications not only severely diminish patients’ quality of life but also impose substantial economic burdens. Studies indicate that annual healthcare costs associated with diabetic complications can rise by more than 50% [4].

Currently, the most commonly prescribed oral hypoglycemic agents for the treatment of T2DM include metformin, acarbose, and glimepiride. Although these drugs are effective in regulating blood glucose levels, their use is frequently associated with adverse effects such as gastrointestinal disturbances and, in some cases, arthralgia [5,6]. Given the lifelong requirement for glycemic management in patients with T2DM, there is increasing interest in the development of natural therapeutic alternatives that can ameliorate clinical symptoms without causing undesirable side effects.

In recent years, the rapid development of microbiomics and metabolomics technologies has significantly advanced our understanding of T2DM. Microbiomics studies have demonstrated a strong association between gut microbiota dysbiosis and the development of T2DM, particularly the reduced abundance of beneficial genera such as Bifidobacterium and Lactobacillus, which correlates with metabolic dysfunction. Concurrently, metabolomic analyses have identified key diabetes-related metabolic biomarkers, including alterations in short-chain fatty acids, bile acids, and branched-chain amino acids, offering new insights into the pathophysiology of the disease. α-Amylase and α-glucosidase are critical enzymes involved in the hydrolysis of dietary carbohydrates into monosaccharides. Inhibition of these enzymes can attenuate glucose absorption, thereby reducing postprandial hyperglycemia. Acarbose is a widely used inhibitor that targets these enzymes [7]. Furthermore, metabolites derived from the gut microbiota can have dual effects on diabetes; short-chain fatty acids (e.g., acetate, propionate, and butyrate) are known to enhance insulin sensitivity, whereas endotoxins such as lipopolysaccharides induce inflammation and aggravate insulin resistance [8].

Although accumulating evidence suggests that probiotics can confer benefits in individuals with T2DM, most studies have yet to identify the specific bioactive components responsible for these effects, and the underlying mechanisms remain poorly understood. In this study, we demonstrated that *Weizmannia coagulans* SA9 significantly reduced blood glucose levels and ameliorates dyslipidemia in diabetic mice. Using high-performance liquid chromatography and thin-layer chromatography, we determined that its metabolites contained active components such as 1-deoxynormycin (DNJ) in the culture supernatant. Furthermore, 16S rRNA amplicon sequencing revealed a notable increase in the abundance of *Lactobacillus reuteri* following SA9 treatment. These results demonstrate that *W. coagulans* SA9 represents a promising probiotic candidate for the management and prevention of T2DM.

## 2. Materials and Methods

### 2.1. Strain Source and Preparation

The strain used in this study, *W. coagulans* SA9, was isolated from naturally fermented mulberry residue and identified by the 16S rRNA gene. This strain has been deposited in the Guangdong Microbial Research Institute Preservation Center.

### 2.2. Preparation and Functions of Cell-Free Supernatant (CFS), Cell-Free Extract (CFE), and Cell Metabolite Supernatant (CMS)

This protocol was adapted from the method described by Ragul K et al. [9]. The strain was first incubated in MRS broth at 37 °C for 48 h. After incubation, the culture was centrifuged, and the supernatant was filtered through a 0.22 μm membrane to obtain the cell-free supernatant (CFS). To prepare the cell-free extract (CFE), the bacterial suspension (10^9^ CFU/mL) was subjected to ultrasonic treatment in an ice bath for 15 min, followed by high-speed centrifugation to remove cell debris, with the resulting supernatant collected. The cell metabolite supernatant (CMS) was prepared by washing the bacterial cells three times with phosphate-buffered saline (PBS, pH 6.8), resuspending them in the same buffer, and adjusting the suspension to 10^9^ CFU/mL. The resuspended cells were incubated at 37 °C with shaking at 180 r/min for 24 h, after which the supernatant was collected by centrifugation.

### 2.3. Assessment of α-Glucosidase Inhibitory Activity

The *p*-nitrophenyl-*α*-D-glucopyranoside (PNPG) assay was conducted as previously described [10]. Briefly, 50 μL of each test sample (CFS, CFE, CMS, or acarbose) was mixed with 100 μL of 5 mM PNPG (Macklin, Shanghai, China) in a 1.5 mL microcentrifuge tube and incubated at 37 °C for 10 min. Subsequently, 100 μL of α-glucosidase (1 U/mL) was added, and the mixture was incubated for an additional 20 min at 37 °C. The reaction was terminated by adding 1 mL of 0.1 M sodium carbonate solution, and the absorbance was measured at 405 nm. Control Group: All conditions were identical, except PBS buffer was used instead of the test sample. Sample Blank Group: All conditions were identical, except 0.1 M PBS buffer replaced α-glucosidase. Blank Group: Both α-glucosidase and the test sample were replaced with PBS buffer.

The α-glucosidase inhibition rate was calculated as follows:

$$Inhibition\;Rate\%=\frac{{(A}_{C}-A_{B})-(A_{S}-A_{SB})}{A_{C}-A_{B}}\times 100\%$$

where *A_C_* is the absorbance value of the control group, *A_B_* is the absorbance value of the sample blank group, *A_S_* is the absorbance value of the sample group, and *A_SB_* is the absorbance value of the sample blank group.

### 2.4. Assessment of α-Amylase Inhibition Capacity

The α-amylase inhibition assay was performed as described by Lee et al. [11]. A 0.5 mL aliquot of each test sample was mixed with 0.5 mL of 1 mg mL^−1^ α-amylase solution and incubated at 37 °C for 10 min. Subsequently, 1 mL of pre-warmed 1.5% (*w*/*v*) soluble starch solution was added, and the mixture was further incubated at 37 °C for 5 min. The reaction was terminated by adding 1 mL 3,5-dinitrosalicylic acid (DNS), followed by heating in a boiling water bath for 5 min. After rapid cooling to room temperature, the mixture was diluted tenfold with distilled water and allowed to stand for 30 min; the absorbance was measured at 540 nm (designated as *A*). Parallel control groups were prepared as follows: B: α-amylase solution replaced with distilled water. C: Test sample replaced with distilled water. D: Both α-amylase solu-tion and test sample replaced with distilled water.

The α-amylase inhibition rate was calculated as follows:

$$Inhibition\;Rate\%=(1-\frac{A-B}{C-D})\times 100\%$$

### 2.5. Untargeted Metabolomics and Active Compound Identification

Thin-layer chromatography (TLC) was employed to detect the presence of 1-DNJ in the samples [12]. The standard and sample solutions were spotted onto a silica gel plate, with chloroform/methanol (1:3, *v*/*v*) as the developing solvent. After development, the solvent front was removed approximately 1 cm from the top of the plate, and the plate was then dried. A color-developing reagent, prepared by mixing 16 mg of o-toluidine, 3 mL of glacial acetic acid, and 0.1 g of potassium iodide, was sprayed onto the plate. The presence of 1-DNJ was qualitatively confirmed by the appearance of purple spots.

High-performance liquid chromatography (HPLC) was conducted for quantitative analysis [13]. Samples were derivatized using 0.4 mol/L borate buffer and FMOC-Cl acetonitrile solution, neutralized with 0.1 mol/L glycine solution, and filtered prior to injection. The chromatographic conditions were as follows: a C18 column, a mobile phase of acetonitrile/acetic acid water (35:65, *v*/*v*), a flow rate of 1.0 mL/min, a column temperature of 30 °C, and a detection wavelength of 254 nm.

Furthermore, non-targeted metabolomics analysis of the fermentation supernatant was performed using an LC-MS platform to identify bioactive metabolites. This analysis was conducted by a professional service provider, Shanghai Majorbio Bio-Pharm Technology Co., Ltd. (Shanghai, China).

### 2.6. Animal and Experimental Design

Six-week-old male db/db mice (C57BLKS/J) and wild-type control mice were provided by the Sterile Animal Platform of Huazhong Agricultural University. During the experiment, the subjects were placed in cages under controlled conditions (22 °C ± 2 °C, 55% ± 5% humidity, 12 h light/dark cycle) with free access to food and water. After 1 week of acclimatization, 8 wild-type db/m mice were used as controls (given saline, Control), and db/db mice were randomly divided into a model group (given saline, T2DM), a *W. coagulans* high-dose treatment group (1 × 10^9^ CFU/g, H-SA9), a *W. coagulans* medium-dose treatment group (given 1 × 10^8^ CFU/g, M-SA9), a *W. coagulans* low-dose treatment group (1 × 10^7^ CFU/g, L-SA9), and a positive drug group (acarbose 50 mg/kg, acarbose), with 8 animals in each group. All treatment groups were administered 0.2 mL of corresponding experimental samples by oral gavage once daily for 6 weeks. All animal procedures were performed in accordance with the “Guidelines for Animal Care and Use of Huazhong Agricultural University” and were approved by the Animal Ethics Committee of the Animal Experiment Center at Huazhong Agricultural University (Approval Number: HZAUMO-2024-0053).

### 2.7. Observation of Urinary Output in Mice

One week before the end of the experiment, we checked the water bottles daily for leaks. By observing the bedding status of the mice, the urination of the mice was recorded in the form of scoring (Table 1).

### 2.8. Oral Glucose Tolerance Test (OGTT) and Insulin Tolerance Test (ITT)

After five weeks of continuous intervention, mice were fasted for 12 h and subjected to an OGTT. Glucose was administered orally at 2 g/kg body weight. Blood samples were collected from the tail vein at 0, 30, 60, 90, and 120 min after gavage, and glucose concentrations were determined with a handheld glucometer (Yuwell 580, Shanghai, China).

Three days after the OGTT, mice were fasted for 4 h, and an ITT was performed. Db/db mice received human insulin at a dose of 2 U/kg, while db/m mice received 1 U/kg. Tail-vein blood was collected 0, 30, 60, 90, and 120 min post-injection, and glucose levels were determined with the same glucometer.

### 2.9. Sample Collection

At the end of the experiment, the mice (fasting for 12 h) were euthanized after inhalation with carbon dioxide. The serum was collected, centrifuged at 4000× *g* for 10 min at 4 °C, and stored at −80 ° C. The contents of the liver, pancreas, colon, and cecum were harvested. Samples were allocated for biochemical analysis (stored at −80 °C), histopathological evaluation (fixed at 4% neutral buffered formalin), and microbiota analysis were conducted.

### 2.10. Analysis of Blood Biochemical Parameters

Enzyme-linked immunosorbent assay (ELISA) kits (Nanjing Jiancheng Bioengineering Institute, Nanjing, China) were used to measure the levels of HbA1c, insulin (Ins), tumor necrosis factor-alpha (TNF-α), interleukin-6 (IL-6), interleukin-10 (IL-10), interleukin-1 beta (IL-1β), lipopolysaccharide (LPS), low-density lipoprotein cholesterol (LDL-C), high-density lipoprotein cholesterol (HDL-C), total cholesterol (TC), and triglycerides (TGs) in the serum according to the manufacturer’s instructions.

### 2.11. Tissue Histopathological Analysis

Pancreatic tissues were fixed in 4% paraformaldehyde, dehydrated in a graded alcohol series, cleared in xylene, and embedded in paraffin. Paraffin-embedded tissue was cut into 5 µm thick sections and rehydrated with xylene followed by a graded alcohol series. For hematoxylin and eosin (H&E) staining, sections were stained for 1–5 min, washed with water, dehydrated with graded alcohol, cleaned with xylene, and mounted in a neutral resin medium.

Immunofluorescence staining of insulin (FITC) and glucagon (CY3) was performed on paraffin sections, following a sequential staining protocol. After dewaxing the samples in water, antigen retrieval was conducted, followed by washing with PBS for 5 min each time, repeated three times on a shaker. The primary antibodies, blocked with 3% BSA at room temperature for 30 min, were incubated overnight at 4 °C and subsequently rinsed with PBS for 5 min each time, also repeated three times. The secondary antibodies were washed at room temperature for one hour and then rinsed three times with PBS for 5 min each. The nuclei were stained with DAPI (37 °C for 10 min in the dark), followed by washing with PBS for a total of three washes of 5 min each. Finally, fluorescence quenching was achieved using a quencher solution (for 5 min), followed by washing with distilled water for an additional 10 min before mounting the slides using an anti-fluorescence quencher tablet.

### 2.12. Gut Microbiota Analysis

Bacterial DNA was extracted from cecal samples, and its purity was assessed using 1% agarose gel electrophoresis. The bacterial 16S rRNA gene V4 region was amplified via PCR using forward and reverse primers. For product detection, an equal volume of 1× loading buffer was combined with the PCR products, which were then electrophoresed on a 2% agarose gel. PCR products within the 400–600 bp range were excised and collected. The samples were pooled based on equal mass concentrations, and a sequencing library was constructed using standard Illumina protocols. The library was sequenced on a MiSeq platform (Illumina, Santiago, CA, USA).

### 2.13. Statistical Analysis

Data are expressed as mean ± SEM. Differences between groups were assessed using one-way ANOVA followed by Dunnett’s multiple comparison tests with Prism 6.0 software (GraphPad Software Inc., San Diego, CA, USA).

## 3. Results

### 3.1. Significant Inhibition of α-Glucosidase and α-Amylase by W. coagulans SA9

**Figure 1:**
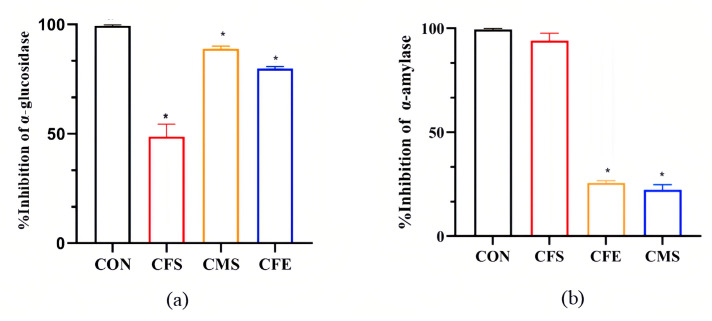
In vitro antidiabetic evaluation. (**a**) Inhibition activity of α-glucosidase. (**b**) Inhibition activity of α-amylase. CON, acarbose; CFS, cell-free supernatant of SA9; CMS, cell metabolite supernatant of SA9; CFE, cell-free extract of SA9. Data were expressed as mean ± SEM. * *p* < 0.05 was regarded as significantly different compared with the CON group.

Carbohydrates constitute the primary energy source for humans, and dietary polysaccharides must be hydrolyzed by glycosidases into monosaccharides for absorption and utilization. Among these enzymes, α-glucosidase and α-amylase play pivotal roles in carbohydrate catabolism, markedly affecting glucose production and absorption [14]. As illustrated in Figure 1, the fractions derived from *W. coagulans* SA9 exhibited distinct inhibitory activities against these two carbohydrate-hydrolyzing enzymes. For α-glucosidase, the inhibition percentages decreased in the following order: CMS (88.90%), CFE (77.54%), and CFS (45.67%). In contrast, inhibition of α-amylase followed the sequence CFS (98.22%), CFE (25.56%), and CMS (22.33%) (Figure S1).

### 3.2. The Metabolic Products of W. coagulans SA9 Contain a Variety of Potent Anti-Hyperglycemic Substances

**Figure 2:**
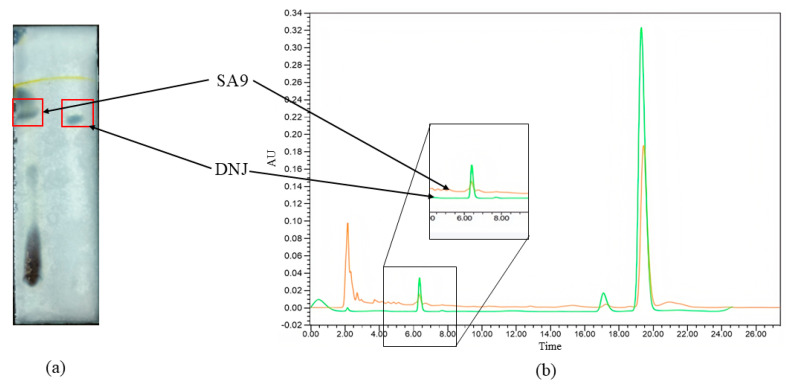
Determination of the suspected active substance DNJ in the fermentation supernatant of SA9. (**a**) TLC. (**b**) HPLC.

As shown in Figure 2a, thin-layer chromatography (TLC) results revealed that the right lane represents the DNJ standard, while the left lane corresponds to the fermentation supernatant of *W. coagulans* SA9. A purple spot appeared in the SA9 supernatant at the same position as the DNJ standard, suggesting the presence of 1-DNJ. This finding was corroborated by high-performance liquid chromatography (HPLC) results (Figure 2b). The green curve represents the DNJ standard, while the orange curve corresponds to the SA9 fermentation supernatant. Both exhibited a retention time of 6.4 min for their first peak, confirming the likely presence of 1-DNJ in the supernatant. We used non-targeted metabolomics to analyze the substances in the supernatant of the culture medium and found that it contained a variety of substances related to the alleviation of diabetes, such as 1-deoxynojirimycin and its derivatives, cinnamic acid and its derivatives, chitin, etc. These compounds are known to exhibit anti-diabetic properties through various mechanisms, such as glycemic control and lipid metabolism regulation.

The metabolites of *W. coagulans* SA9 include 1-DNJ and other bioactive compounds, providing a molecular basis for its potential anti-diabetic effects. Further investigation into the mechanisms of these metabolites could reveal novel therapeutic applications.

### 3.3. Positive Effects of W. coagulans SA9 on Diabetic Symptoms in db/db Mice

**Figure 3:**
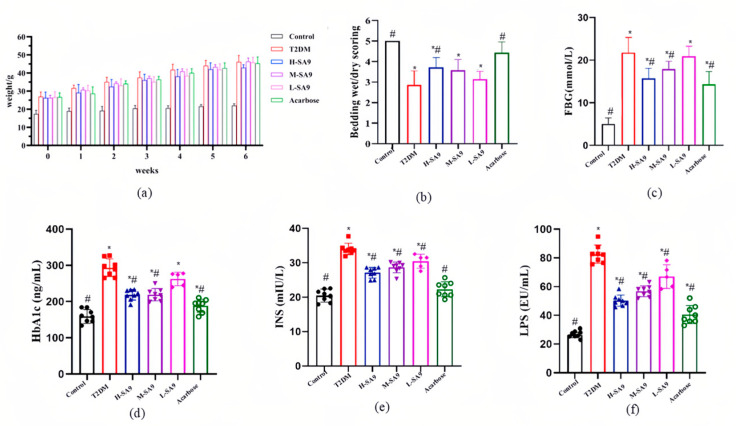
Coagulation of *W. coagulans* stops T2DM from worsening (*n* = 8). (**a**) Weight. (**b**) Urinary output. (**c**) Fasting blood glucose. (**d**) HbAlc. (**e**) INS. (**f**) LPS. Control, normal group; T2DM, diabetes model; H-SA9, 10^9^ CFU/day of SA9; M-SA9, 10^8^ CFU/day of SA9; L-SA9, 10^7^ CFU/day of SA9. Data were expressed as mean ± SEM. * *p* < 0.05 was regarded as significantly different compared with the Control group, and # *p* < 0.05 was regarded as significantly different compared with the T2DM group.

Due to a leptin receptor mutation, db/db mice develop obesity and hyperglycemia beginning at approximately three weeks of age [15]. The body weight trajectory of db/db mice is shown in Figure 3a. With increasing age, these mice exhibit rapid weight gain, and probiotic administration does not exert a significant effect on weight control. Notably, *W. coagulans* SA9 alleviated diabetic symptoms, as evidenced by a marked reduction in polyuria (Figure 3b, Figure S2), and fasting blood glucose levels in the probiotic group were significantly lower than that in the T2DM group (Figure 3c). Furthermore, compared with the T2DM group, both the H-SA9 and M-SA9 groups showed significant reductions (*p* < 0.05) in HbA1c, serum insulin, and LPS levels; specifically, the H-SA9 group exhibited decreases of 25.01%, 20.38%, and 38.96%, respectively (Figure 3d–f).

**Figure 4:**
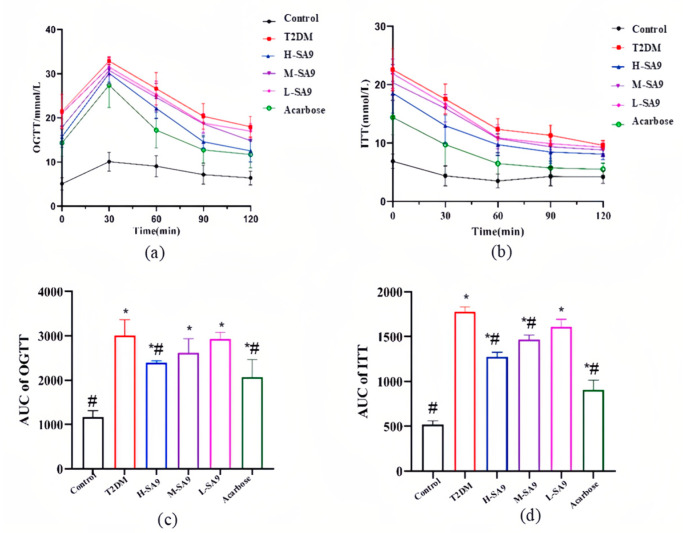
SA9 improves glucose tolerance levels in mice (*n* = 8). (**a**) OGTT. (**b**) ITT. (**c**) AUC of OGTT. (**d**) AUC of ITT. Control, normal group; T2DM, diabetes model; H-SA9, 10^9^ CFU/day of SA9; M-SA9, 10^8^ CFU/day of SA9; L-SA9, 10^7^ CFU/day of SA9. Data were expressed as mean ± SEM. * *p* < 0.05 was regarded as significantly different compared with the Control group, and # *p* < 0.05 was regarded as significantly different compared with the T2DM group.

Glucose tolerance and insulin resistance were evaluated through glucose tolerance tests (GTTs) and insulin tolerance tests (ITTs). In the GTTs, blood glucose levels in all groups, except for the normal control group, rose sharply after the administration of a 20% glucose solution and peaked at 30 min (Figure 4a). Intervention with *W. coagulans* SA9 resulted in a dose-dependent reduction in AUC values, with the high-dose (H) group exhibiting a significant 20.41% reduction compared to the DM group (Figure 4c). In the ITTs, blood glucose levels decreased over time following intraperitoneal injection of rapid-acting insulin (Figure 4b). AUC values in the H and medium-dose (M) groups were significantly reduced compared to the DM group (*p* < 0.05; Figure 4d), indicating improved insulin sensitivity.

### 3.4. W. coagulans SA9 Alleviates Dyslipidemia in db/db Mice

**Figure 5:**
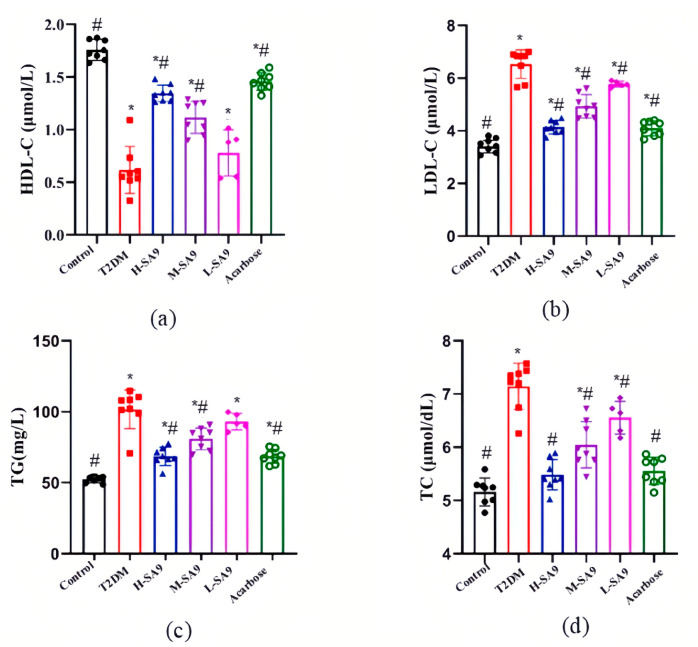
Effect of SA9 on lipid tetrads in db/db mice (*n* = 8). (**a**) HDL-C. (**b**) LDL-C. (**c**) TG. (**d**) TC. Control, normal group; T2DM, diabetes model; H-SA9, 10^9^ CFU/day of SA9; M-SA9, 10^8^ CFU/day of SA9; L-SA9, 10^7^ CFU/day of SA9. Data were expressed as mean ± SEM. * *p* < 0.05 was regarded as significantly different compared with the Control group, and # *p* < 0.05 was regarded as significantly different compared with the T2DM group.

The levels of HDL-C, LDL-C, TG, and TC in the experimental groups are presented in Figure 5. Diabetic mice (T2DM group) exhibited significantly increased TG, TC, and LDL-C levels and decreased HDL-C levels compared to the Control group (*p* < 0.05).

After 6 weeks of intervention with *W. coagulans* SA9, the lipid profiles of diabetic mice improved markedly. In the H-SA9 group, TG, TC, and LDL-C levels were reduced by 33.94%, 23.24%, and 40.18%, respectively, while HDL-C levels increased by 113.12% compared to the DM group. In the L-SA9 group, TG, TC, and LDL-C levels were reduced by 10.17%, 8.26%, and 9.73%, respectively, and HDL-C increased by 32.75%. Statistically significant differences (*p* < 0.05) in TG, TC, and LDL-C levels were observed between the T2DM group and the H-SA9 and M-SA9 groups. However, in the L-SA9 group, only TC and LDL-C levels showed significant differences (*p* > 0.05). These findings suggest that *W. coagulans* SA9 can effectively ameliorate dyslipidemia, thereby contributing to the restoration of lipid homeostasis and improved blood lipid profiles in diabetic mice.

### 3.5. W. coagulans SA9 Alleviates Systemic Inflammation in db/db Mice

**Figure 6:**
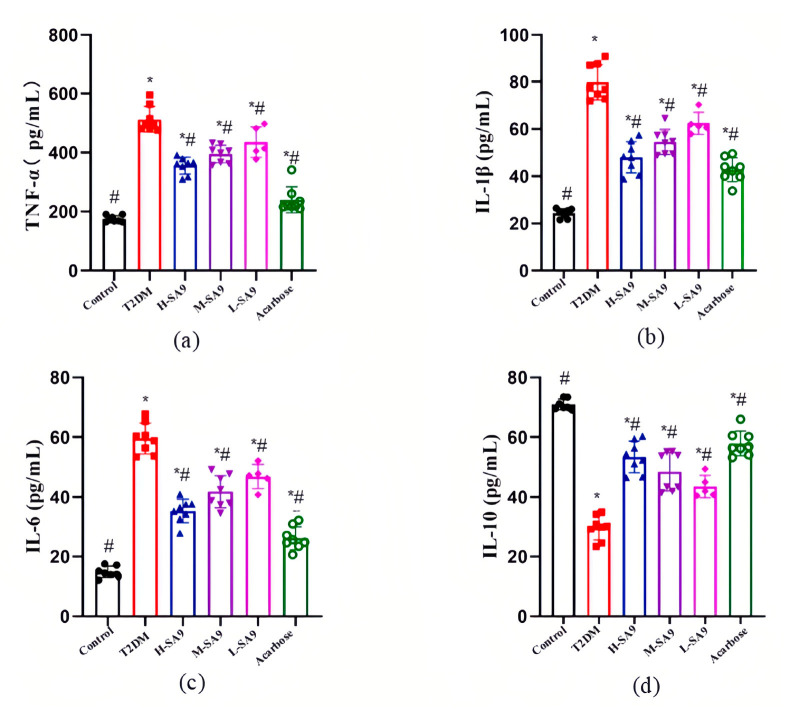
Effects of *W. coagulans* SA9 on inflammation-related factors in the db/db mouse model of T2DM (*n* = 8). (**a**) TNF-α. (**b**) IL-1β. (**c**) IL-6. (**d**) IL-10. Control, normal group; T2DM, diabetes model; H-SA9, 10^9^ CFU/day of SA9; M-SA9, 10^8^ CFU/day of SA9; L-SA9, 10^7^ CFU/day of SA9. Data were expressed as mean ± SEM. * *p* < 0.05 was regarded as significantly different compared with the Control group, and # *p* < 0.05 was regarded as significantly different compared with the T2DM group.

To evaluate the anti-inflammatory effects of *W. coagulans* SA9, we measured the levels of TNF-α, IL-6, IL-10, IL-1β, and endotoxins. The results demonstrate that *W. coagulans* SA9 intake significantly attenuated systemic inflammation (Figure 6). At the end of the experiment, diabetic mice in the T2DM group exhibited elevated levels of pro-inflammatory cytokines IL-1β, TNF-α, and IL-6, along with reduced levels of the anti-inflammatory cytokine IL-10. Treatment with *W. coagulans* SA9 improved these parameters, as evidenced by increased IL-10 levels and decreased levels of IL-1β, TNF-α, and IL-6. Specifically, in the H-SA9 group, IL-1β, TNF-α, and IL-6 levels decreased by 39.97%, 42.26%, and 31.30%, respectively, while IL-10 levels increased by 81.13% compared with the T2DM group. These findings suggest that *W. coagulans* SA9 modulates immune function by downregulating pro-inflammatory cytokines and enhancing anti-inflammatory responses, thereby alleviating systemic inflammation.

### 3.6. W. coagulans SA9 Protects Islet Structure in db/db Mice

**Figure 7:**
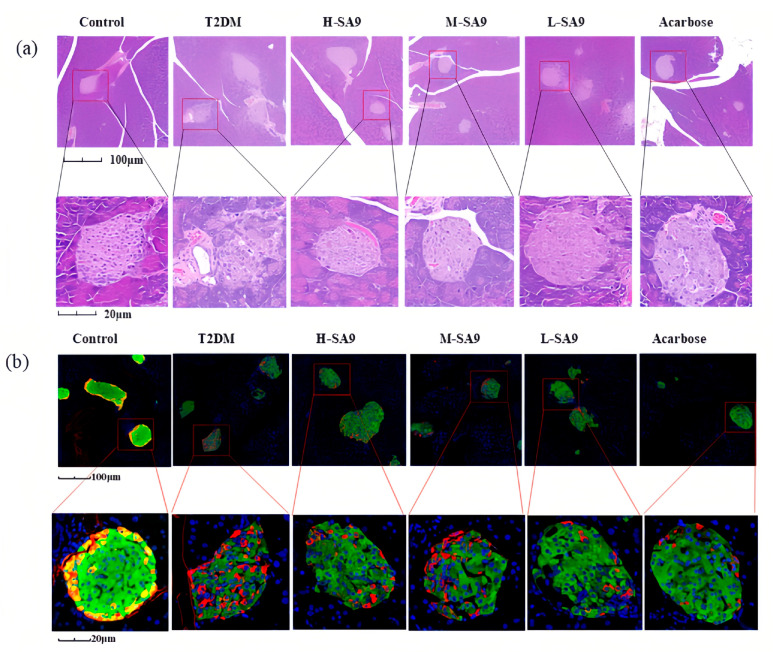
Protective effect of *W. coagulans* SA9 on pancreatic islets (*n* = 6). (**a**) HE-stained section of the pancreas of db/db mice (400×). (**b**) IF-stained section of the pancreas of db/db mouse (400×). Insulin-positive cells are stained red, glucagon-positive cells are stained green, and nuclei are stained blue. Control, normal group; T2DM, diabetes model; H-SA9, 10^9^ CFU/day of SA9; M-SA9, 10^8^ CFU/day of SA9; L-SA9, 10^7^ CFU/day of SA9.

Histological examination revealed well-defined, round, or oval islets interspersed among acinar cells in healthy mice. In the T2DM group, islets were markedly atrophic and deformed with ill-defined margins (Figure 7a, Figure S3). Treatment with *W. coagulans* SA9 or acarbose clearly mitigated islet deterioration. Immunofluorescence staining demonstrated a pronounced expansion of α-cell area in diabetic mice, whereas probiotic treatment significantly increased β-cell area, indicating that *W. coagulans* SA9 effectively suppresses hyperglucaginemia and preserves pancreatic function (Figure 7b, Figure S4).

### 3.7. Regulatory Effects of W. coagulans SA9 on the Gut Microbiota of db/db Mice

**Figure 8:**
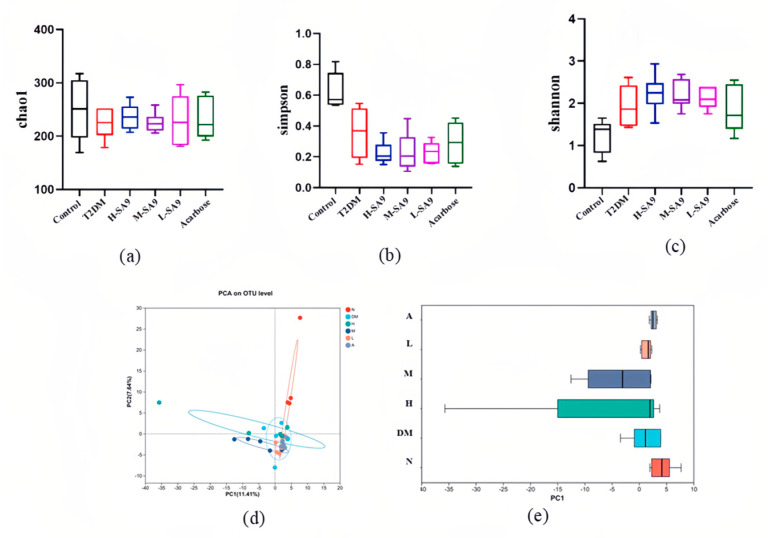
Effect of *W. coagulans* SA9 on the structure of intestinal microorganisms (*n* = 6). (**a**) Chao1. (**b**) Simpson. (**c**) Shannoon. (**d**) PCoA. (**e**) Boxplot. Control, normal group; T2DM, diabetes model; H-SA9, 10^9^ CFU/day of SA9; M-SA9, 10^8^ CFU/day of SA9; L-SA9, and 10^7^ CFU/day of SA9. Data were expressed as mean ± SEM.

**Figure 9:**
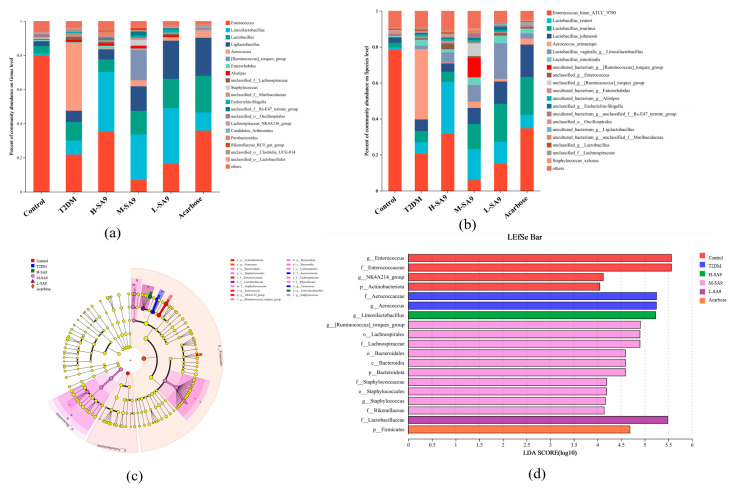
Modulation of gut microorganisms by *W. coagulans* SA9 (*n* = 6). (**a**) Genus level. (**b**) Species level. (**c**) LEfSe analyzing cluster trees. (**d**) LDA analysis histogram. Control, normal group; T2DM, diabetes model; H-SA9, 10^9^ CFU/day of SA9; M-SA9, 10^8^ CFU/day of SA9; L-SA9, and 10^7^ CFU/day of SA9. Data were expressed as mean ± SEM.

The gut microbiota plays a pivotal role in metabolic regulation and is closely associated with the pathogenesis of diabetes and obesity [16]. In this study, we investigated the effects of *W. coagulans* SA9 on the composition and structure of the intestinal flora of db/db mice. In Alpha Diversity, we used Chao1, Simpson and Shannon index indices to assess species diversity (Figure 8a–c), and found that the Simpson index of the probiotic group decreased significantly but not statistically significant (*p* < 0.05) indicating that SA9 has a limited effect on gut microbial diversity. Regarding beta diversity (Figure 8d,e), principal coordinate analysis (PCoA) revealed substantial differences in microbial community structure between the probiotic M-SA9 and T2DM groups. The microbial community structure of the probiotic L-SA9 and the acarbose groups was similar to that of the T2DM group. To elucidate microbiota-mediated mechanisms, we analyzed the gut communities of the Control, T2DM, high-dose SA9 (H-SA9), medium-dose SA9 (M-SA9), and low-dose SA9 (L-SA9) groups. At both the genus and species levels, the relative abundance of *Limosilactobacillus reuteri* was significantly elevated in probiotic-treated mice. LefSe cladograms (Figure 9a) and LDA bar plots (Figure 9b) highlighted distinct signature taxa: *Aerococcaceae*/*Aerococcus* in the T2DM group; *Limosilactobacillus* in H-SA9; multiple taxa including *Lachnospiraceae*, *Bacteroidota*, and Staphylococcus in M-SA9; *Lactobacillaceae* in L-SA9; and Firmicutes in the acarbose group. These findings suggest that *W. coagulans* SA9 alleviates diabetic phenotypes by reshaping the intestinal microbiota.

## 4. Discussion

The concept of “live biotherapeutics” has emerged alongside advances in gut microbiota research. These active microbial preparations, comprising either living microorganisms or substances that promote microbial growth, have demonstrated considerable potential in the prevention, treatment, and alleviation of various diseases [17]. Given the alarming prevalence of T2DM and its associated complications, the identification of safe and effective intervention strategies is of critical importance. In this study, we identified a strain, *W*. *coagulans* SA9 that produces potent anti-glycemic substances such as DNJ, which can effectively ameliorate diabetic symptoms. These findings provide new perspectives and empirical evidence supporting the use of probiotics in the management of diabetes.

*W. coagulans* is a moderately thermophilic bacterium with an optimal growth temperature of 35 °C to 50 °C. The bacterium grows at a maximum temperature of 57 °C to 60° [18]. This species has been well documented for its ability to alleviate conditions such as non-alcoholic fatty liver disease [19], constipation [20], and diarrhea [21] and is considered to be an ideal probiotic candidate. Carbohydrates ingested by the human body need to be broken down into glucose under the action of digestive enzymes such as α-glucosidase and α-amylase before they can be absorbed and utilized by the body. Therefore, inhibiting the activity of these digestive enzymes is one of the most important measures to prevent postprandial hyperglycemia. Oral hypoglycemic drugs developed according to this principle are called α-glucosidase inhibitors, such as acarbose and voglibose. A large amount of evidence indicates that probiotics are potential inhibitors of α-glycosidase and exhibit inhibitory effects on both α-glucosidase and α-amylase activities [22]. For instance, Lactobacillus strain 21828, isolated from apple juice, showed 35.00% α-glucosidase inhibition [23], while strains of Lactobacillus plantarum and *Lactobacillus paracasei* demonstrated α-amylase inhibition exceeding 85% [24]. Our in vitro analysis of *W. coagulans* SA9 revealed that its CME had the highest α-glucosidase inhibitory activity, while CFS was most effective against α-amylase. The CFE of *W. coagulans* SA9 exhibited strong inhibitory effects on α-glucosidase but weak inhibitory capacity against α-amylase, similar to the results of Frediansyah et al. [25], who attributed such variations to strain-specific metabolic products. These results highlight the potential of *W. coagulans* SA9 as a live biotherapeutic agent for managing diabetes and its associated metabolic disorders. Further research is needed to explore the mechanisms underlying its strain-specific effects.

The functional properties of probiotics are fundamentally dependent on their specific material basis. In this study, we further investigated the bioactive products of *W. coagulans* SA9 and identified a range of hypoglycemic metabolites, including DNJ [26], cinnamic acid [27], and phaseollin [28]. Among them, phaseollin was of particular interest, as previous studies have demonstrated its efficacy in inhibiting the activity of animal-derived α-amylase [28]. This finding may explain the differential inhibitory effects of various SA9 components on α-glucosidase and α-amylase activity. Microbial-derived α-glycosidase inhibitors are known to be structurally complex and diverse. For instance, Chandrasekhar et al. isolated 1-O-methyl chrysophanol from Thermo-actinomyces vulgaris SFMA-103, which inhibited carbohydrate-metabolizing enzymes with IC50 values of 38.49 μg/mL (α-glucosidase) and 3.4 mg/mL (α-amylase) [29]. Similarly, Kang et al. identified a tripeptide Pro-Phe-Pro, molecular weight 360.1 Da) from *Aspergillus oryzae* N159-1 as an α-glucosidase inhibitor through LC-MS/MS analysis [30]. Although the untargeted metabolic analysis of this study revealed multiple hypoglycemic bioactive compounds, further research is required to elucidate which substance plays the predominant role.

We further evaluated the anti-diabetic potential of *W. coagulans* SA9 in vivo. Currently, there is no ideal animal model that fully replicates the pathophysiological features of human T2DM. The db/db mouse, one of the earliest and most extensively studied models of diabetes, carries a mutation in the leptin receptor, resulting in hyperphagia, pronounced obesity, and insulin resistance beginning at three weeks of age, with persistently elevated blood glucose levels [31]. Growing evidence indicates that probiotics are playing an increasingly important role in improving type 2 diabetes. Wang et al. revealed the role of *Lactobacillus plantarum* SHY130 in improving diabetes, which can enhance insulin resistance and increase glucose tolerance [32]. In the present study, *W. coagulans* SA9 intervention yielded striking results in db/db mice. Administration of *W. coagulans* SA9 led to decreased food and water intake, reduced glycated hemoglobin levels, and improved polyuria. These findings suggest that *W. coagulans* SA9 can effectively ameliorate key symptoms and metabolic dysfunctions associated with T2DM in db/db mice.

Dyslipidemia is a key risk factor for T2DM and is characterized by decreased HDL-C levels, increased LDL-C levels, and abnormal TC and TG levels. HDL-C facilitates the removal of excess cholesterol from blood vessels, whereas LDL-C transports cholesterol to tissues, with elevated LDL-C levels contributing to atherosclerosis [33]. Abnormal TC and TG levels exacerbate insulin resistance, increasing the risk of T2DM. Notably, dyslipidemia often manifests during the prediabetic stage [34]. Dyslipidemia also plays a pivotal role in the progression of diabetes complications. Even with controlled blood glucose levels, dyslipidemia can lead to diabetic nephropathy, neuropathy, and retinopathy [35]. Evidence suggests that probiotics can modulate dyslipidemia in diabetic mice, offering a potential therapeutic approach [36]. T2DM is further exacerbated by chronic low-grade inflammation, which contributes to the progression of diabetes and its complications. Inflammation activates pathways such as NF-κB and JNK, elevating pro-inflammatory cytokines like TNF-α and IL-6. These cytokines disrupt the IRS-1/PI3K/AKT signaling pathway by inhibiting IRS-1 phosphorylation, impairing glucose metabolism, and exacerbating insulin resistance [37]. In addition, inflammation promotes endothelial dysfunction and oxidative stress, accelerating the development of diabetic complications, with lipopolysaccharide (LPS) serving as a key inflammatory trigger [38]. Anti-inflammatory cytokines such as IL-10 mitigate these effects by inhibiting NF-κB activation, reducing pro-inflammatory cytokine production, and promoting the anti-inflammatory polarization of macrophages [39]. Probiotics have been shown to decrease pro-inflammatory cytokines, increase IL-10 levels, alleviate islet damage, and improve insulin resistance [40]. In this study, *W. coagulans* SA9 reduced inflammation in db/db mice, consistent with findings from Zeng et al. [41]. The SA9-treated groups exhibited significant reductions in TNF-α and IL-6 levels (*p* < 0.05). These results underscore the anti-inflammatory potential of *W. coagulans* SA9 and its relevance in managing the inflammation-associated complications of T2DM.

Gut microbiota dysbiosis is a major contributor to the development of diabetes. Over the past decade, research has underscored the essential role of gut microbiota in the pathogenesis of obesity and T2DM [16,42]. In this study, at the genus level, we hypothesized that *W*. *coagulans* SA9 could exert its hypoglycemic effect by increasing the genus *Limosilactobacillus* and reducing the abundance of the genus *Aerococcus*. It is worth noting that at the species level, the abundance of *Limosilactobacillus reuteri* in the probiotic intervention group increased and was dose dependent. Previous studies have demonstrated in randomized controlled trials that *L. reuteri* stimulates the secretion of GLP-1 and GLP-2, thereby improving glucose tolerance. GLP-1 enhances insulin secretion, suppresses glucagon release, and delays gastric emptying, while GLP-2 improves intestinal barrier function and nutrient absorption, indirectly supporting glucose metabolism [43]. *L. reuteri* is a short-chain fatty acid (SCFA)-producing bacterium that regulates gene expression and signal transduction by activating G-protein-coupled receptors (GPRs) and inhibiting histone deacetylases (HDACs). These mechanisms influence both insulin secretion and sensitivity, contributing to metabolic improvements [44]. In addition, *W. coagulans* was not detected at the species level, suggesting that this strain is unable to colonize the intestine, a finding consistent with the results of Adami et al. [45].

A limitation of this study is that the key mechanisms underlying the anti-glycemic effects of *W. coagulans* SA9 remain to be elucidated, and the specific metabolites responsible for these effects have yet to be identified. Additionally, alterations in endogenous gut metabolites were not assessed, as the primary focus was placed on phenotype improvement. In future work, we will employ a range of animal models, as well as advanced histological techniques, to further investigate the antidiabetic effect of *W. coagulans* SA9.

## 5. Conclusions

In conclusion, this study identified and characterized *W. coagulans* SA9, a strain isolated from mulberries, which effectively inhibits α-glucosidase and α-amylase activities and produces multiple anti-diabetic active compounds. The strain was demonstrated to enhance lipid and glucose metabolism, preserve pancreatic islet function, and alter gut microbiome in a T2DM animal model, thereby mitigating key metabolic and clinical features of T2DM. These findings provide a valuable reference for the development of live biotherapeutics targeting T2DM and underscore the potential of *W. coagulans* SA9 as a promising candidate for further research and clinical application.

## 6. Patents

Patents 1: A strain of *Bacillus coagulans* capable of inhibiting alpha-glucosidase and/or alpha-amylase activity and its application.

Patents 2: *Weizmannia coagulans* in the preparation of 1-deoxynojirimycin and in the amelioration of abnormalities of glycolipid metabolism in the body.

## Figures and Tables

**Table 1:** Mouse bedding dry/wet criteria.

Mouse Bedding	Scores
Dry	5
Mostly dry	4
Partly wet	3
Mostly wet	2
Wet	1

## Data Availability

The original contributions presented in this study are included in the article; further inquiries can be directed to the corresponding authors.

## Supplementary Materials

The following supporting information can be downloaded at https://www.mdpi.com/article/10.3390/nu17132081/s1, Figure S1: The ability of SA9 to inhibit the activity of α -amylase was evaluated by the Oxford method; Figure S2: Photos of mice bedding after the fifth week of intervention; Figure S3: The original image of pancreatic immunofluorescence; Figure S4: The original image of HE pancreatic.
