# Supplementary material for: Weizmannia coagulans SA9: A Novel Strategy to Alleviate Type 2 Diabetes
Source: Nutrients. 2025 Jun 23;17(13):2081. doi: 10.3390/nu17132081 (PMC12251212; doi:10.3390/nu17132081)
Supplement: Supplementary file 1 [file nutrients-17-02081-s001.zip › nutrients-3646430-supplementary.pdf]

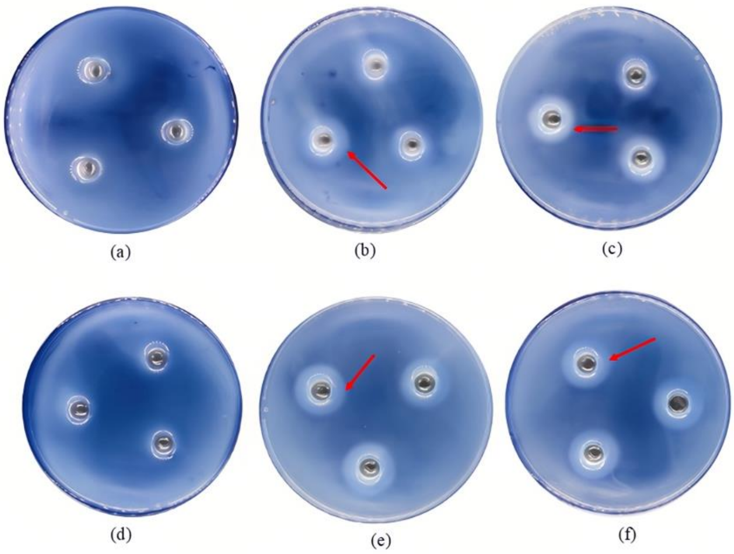

Figure S1. The ability of SA9 to inhibit the activity of α -amylase was evaluated by the Oxford method. (**a**) CFS. (**b**) CFE. (**c**) CMS. (**d**) Acarbose. (**e**) pH 3.0 lactate. (**f**) PBS. CON, Acarbose; CFS, Cell-Free Supernatant SA9; CMS, Cell Metabolite Supernatan*t* of SA9; CFE, Cell-Free Extract of SA9.

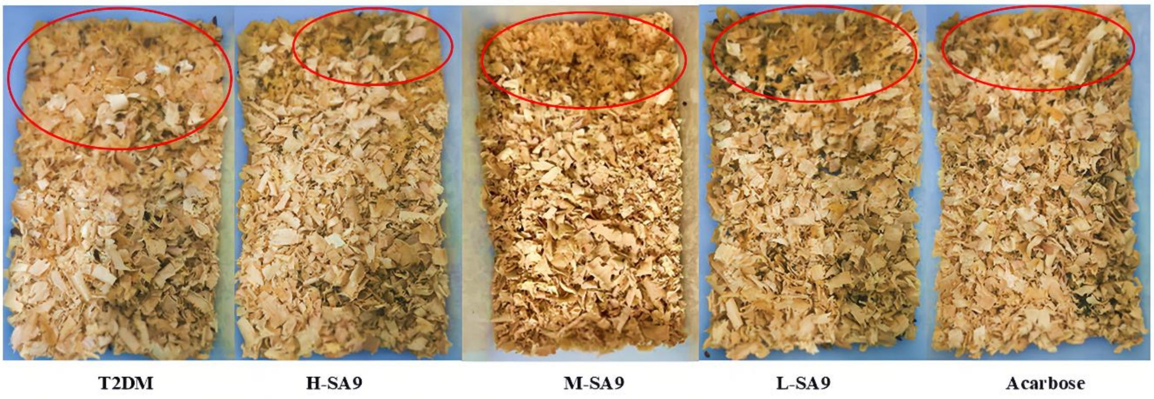

Figure S2. Photos of mice bedding after the fifth week of intervention. Control, Normal group; T2DM, Diabetes model; H-SA9, 10^9 CFU/day of SA9; M-SA9, 10^8 CFU/day of SA9; L-SA9, 10^7 CFU/day of SA9.

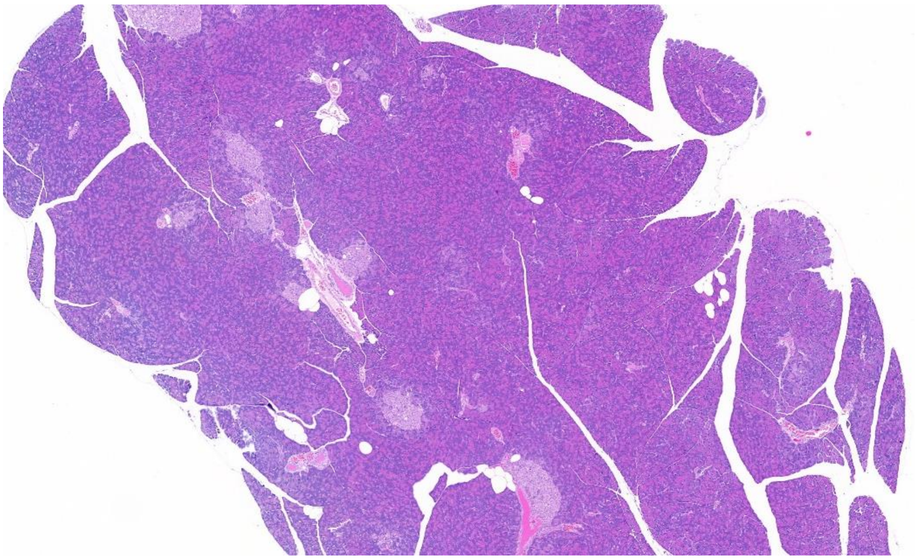

(a) HE the T2DM group(5x)

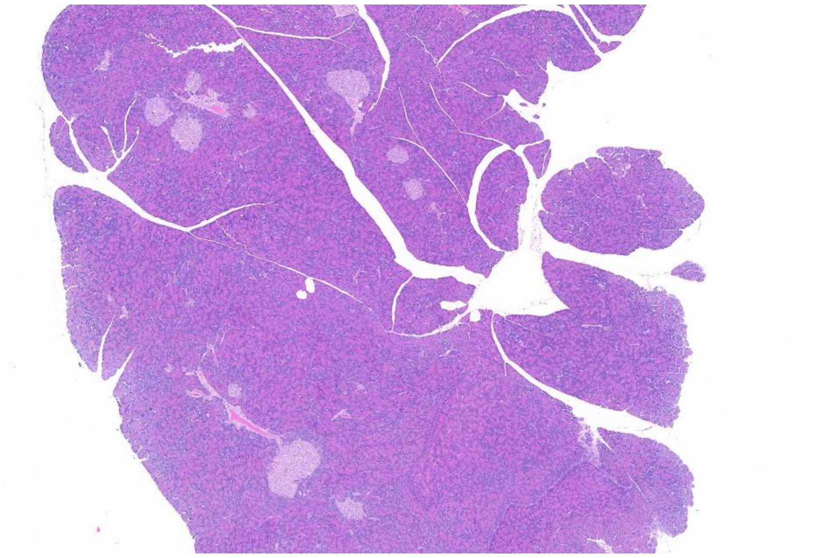

(b) HE the H-SA9 group(5x)

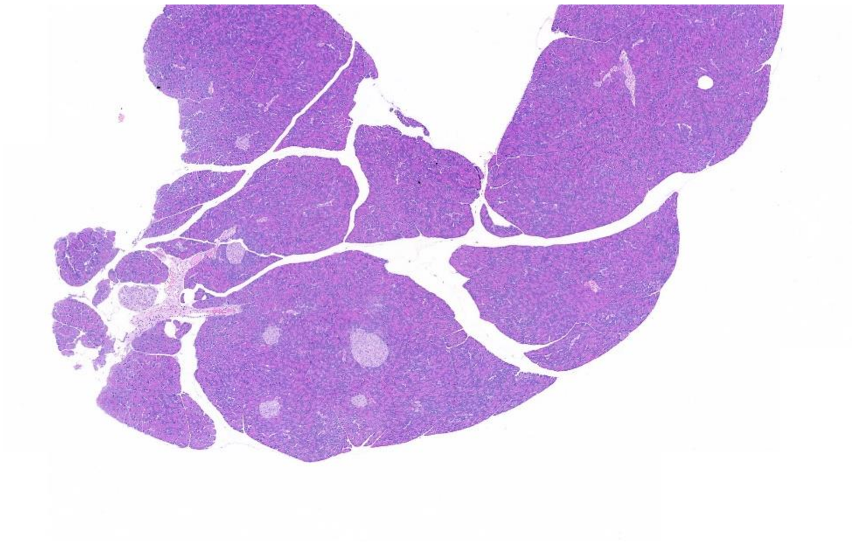

(c) HE the M-SA9 group(5x)

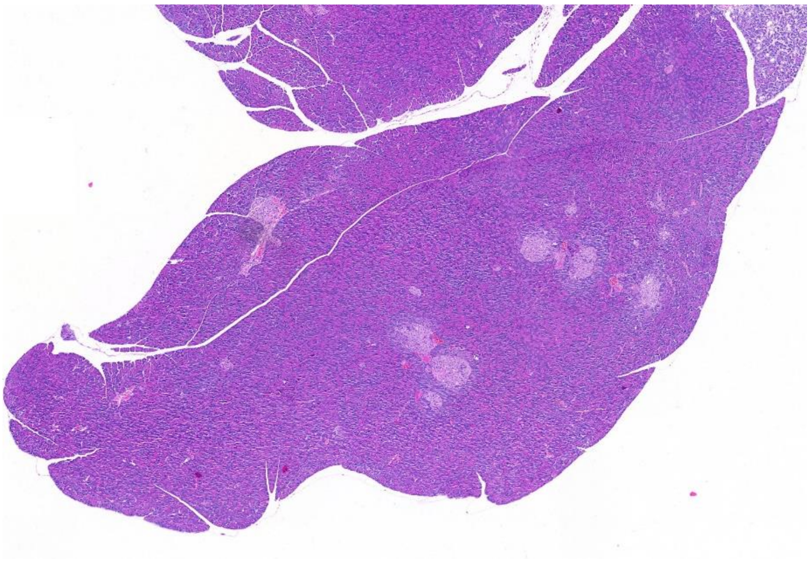

(d) HE the L-SA9 group(5x)

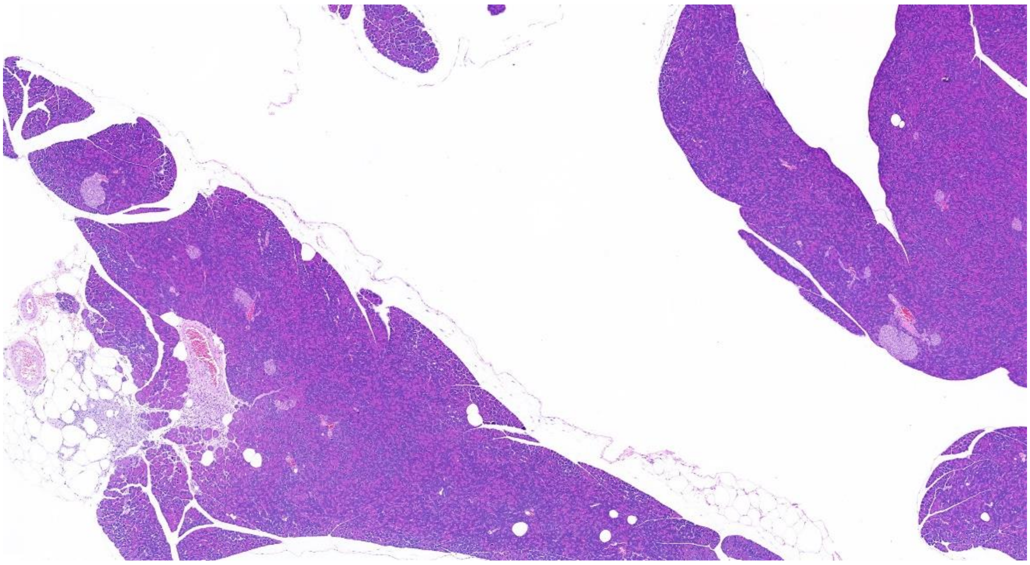

(e) HE the Acarbose group(5x)

Figure S3. The original image of HE pancreatic

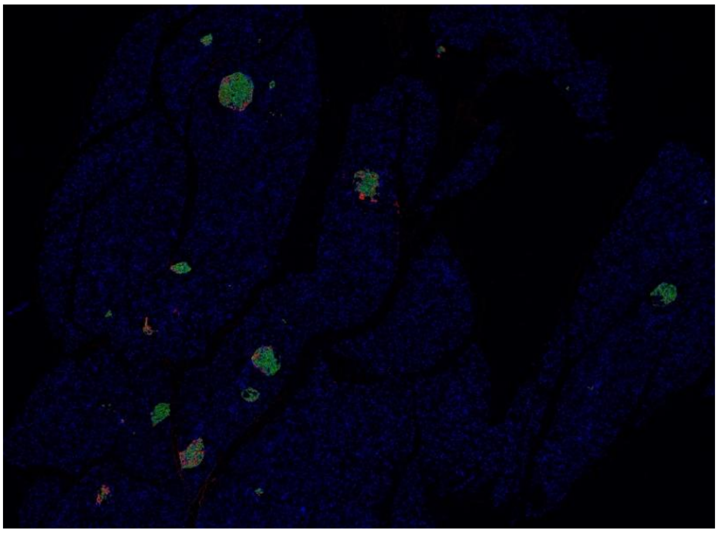

(a) IF the T2DM group (5x)

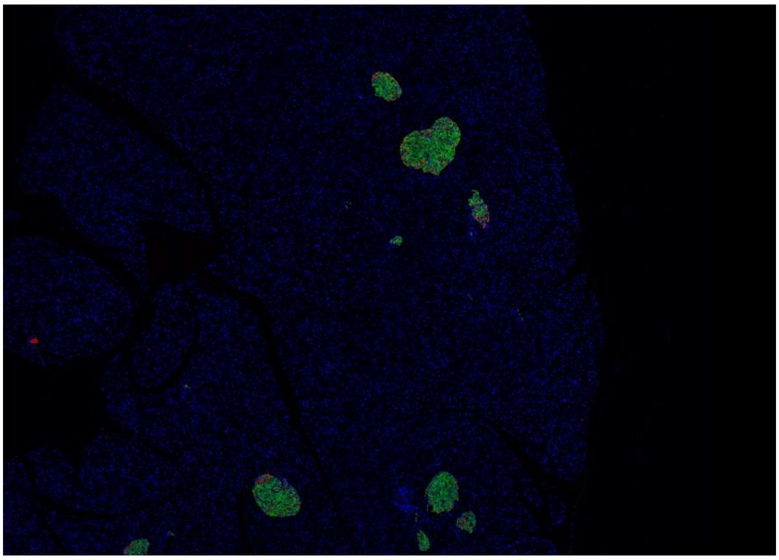

(b) IF the H-SA9 group(5x)

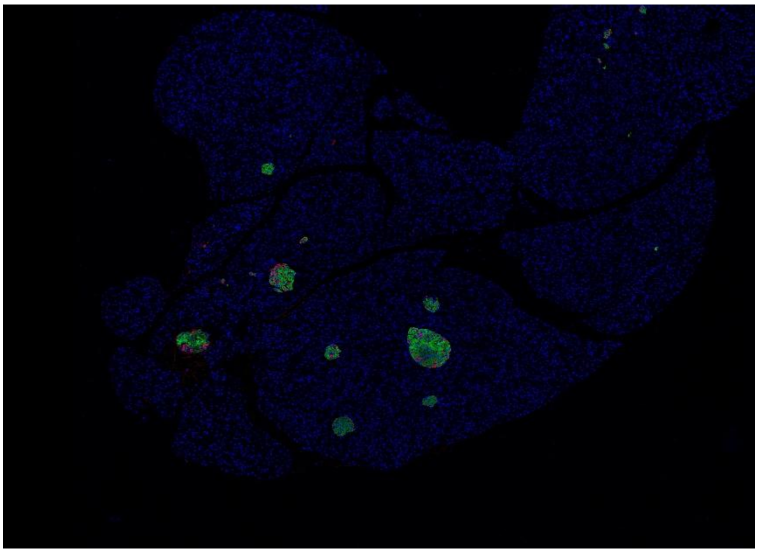

(c) IF the M-SA9 group(5x)

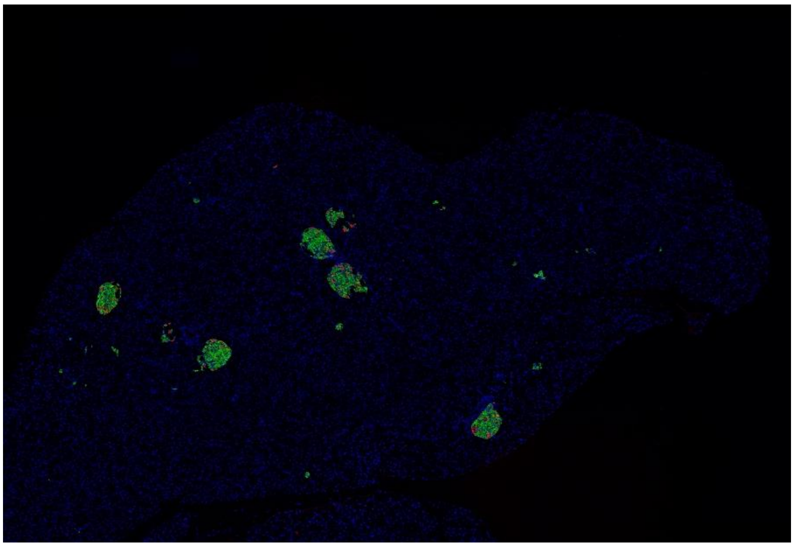

(d) IF the L-SA9 group(5x)

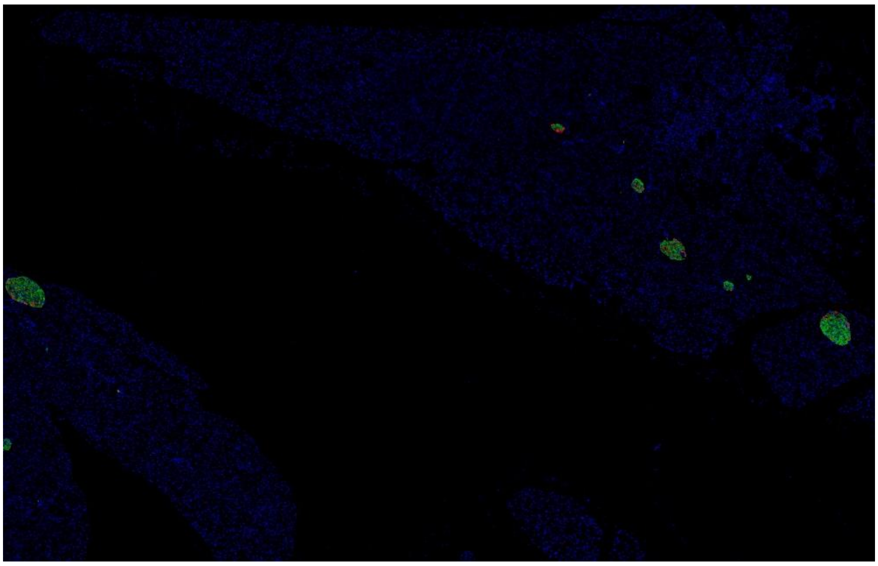

(e) IF the Acarbose group(5x)

Figure S4. The original image of pancreatic immunofluorescence
